# Effect of different surgical type of coronary artery bypass grafting on kidney injury: A propensity score analysis: Erratum

**DOI:** 10.1097/MD.0000000000009527

**Published:** 2017-12-29

In the article, “Effect of different surgical type of coronary artery bypass grafting on kidney injury: A propensity score analysis”^[1]^, which appeared in Volume 96, Issue 45 of *Medicine*, affiliations a and b are missing Chang Gung Memorial Hospital. They should appear as:

a Kidney Research Center, Department of Nephrology, Chang Gung Memorial Hospital, b Department of Cardiothoracic and Vascular Surgery, Chang Gung Memorial Hospital
